# Tumor-derived exosomal long noncoding RNA LINC01133, regulated by Periostin, contributes to pancreatic ductal adenocarcinoma epithelial-mesenchymal transition through the Wnt/β-catenin pathway by silencing AXIN2

**DOI:** 10.1038/s41388-021-01762-0

**Published:** 2021-04-06

**Authors:** Yang Liu, Tianchi Tang, Xiaosheng Yang, Peng Qin, Pusen Wang, Huiping Zhang, Min Bai, Rong Wu, Fan Li

**Affiliations:** 1 Department of Ultrasound, Shanghai General Hospital, Shanghai Jiaotong University School of Medicine, Shanghai, China; 2 Department of Neurosurgery, Affiliated Xinhua Hospital, Shanghai Jiaotong University School of Medicine, Shanghai, China; 3 Department of Neurosurgery, Shanghai Ninth People’s Hospital, Shanghai Jiao Tong University School of Medicine, Shanghai, China; 4 Department of Instrument Science and Engineering, Shanghai Jiao Tong University, Shanghai, China; 5 Department of General Surgery, Shanghai General Hospital, Shanghai Jiao Tong University School of Medicine, Shanghai, China; 6 Department of Ultrasound, Shanghai Changning Maternity and Infant Health Hospital/Maternity and Infant Health Hospital affiliated East China Normal University, Shanghai, China

**Keywords:** Cancer microenvironment, Extracellular matrix, siRNAs

## Abstract

Pancreatic ductal adenocarcinoma (PDAC) is one of the most fatal malignancies and rapidly progressive diseases. Exosomes and long noncoding RNAs (lncRNAs) are emerging as vital mediators in tumor cells and their microenvironment. However, the detailed roles and mechanisms of exosomal lncRNAs in PDAC progression remain unknown. Here, we aimed to clarify the clinical significance and mechanisms of exosomal lncRNA 01133 (LINC01133) in PDAC. We analyzed the expression of LINC01133 in PDAC and found that exosomal LINC01133 expression was high and positively correlated with higher TNM stage and poor overall survival rate of PDAC patients. Further research demonstrated that Periostin could increase exosome secretion and then enhance LINC01133 expression. In addition, Periostin increased p-EGFR, p-Erk, and c-myc expression, and c-myc could bind to the LINC01133 promoter region. These findings suggested that LINC01133 can be regulated by Periostin via EGFR pathway activity. We also observed that LINC01133 promoted the proliferation, migration, invasion, and epithelial–mesenchymal transition (EMT) of pancreatic cancer cells. We subsequently evaluated the effect of LINC01133 on the Wnt/β-catenin pathway and confirmed that LINC01133 can interact with Enhancer Of Zeste Homolog 2 (EZH2) and then promote H3K27 trimethylation. This can further silence AXIN2 and suppress GSK3 activity, ultimately activating β-catenin. Collectively, these data indicate that exosomal LINC01133 plays an important role in pancreatic tumor progression, and targeting LINC01133 may provide a potential treatment strategy for PDAC.

## Introduction

Pancreatic cancer remains one of most extremely aggressive human malignancies. More than 90% of pancreatic cancers are classified as pancreatic ductal adenocarcinoma (PDAC) [1], with the postoperative 5-year survival rate less than 5% [2]. The cause of high mortality in PDAC patients is critically ascribed to the early distant metastases at the time of diagnosis. Among numerous factors, the epithelial-mesenchymal transition (EMT) is an important process that contributes to metastasis [3]. Therefore, identification of the key regulators that enhance PDAC EMT is vital for improving diagnosis and treatment regimens [4].

PDAC is characterized by a prominent desmoplastic reaction [5–7], and EMT usually closely corelates with the microenvironment in which there is abundant infiltration of stromal contents, especially pancreatic stellate cells (PSCs) [8–10]. Periostin, a 90 kilodalton (kD) secretory protein, is specifically secreted by PSCs [11]. Periostin expression in PDAC tumors is 42-fold higher at the mRNA level than in normal pancreas samples [12]. Previously, we have shown that Periostin is a powerful driving force for PDAC pathogenesis [13].

Long noncoding RNAs (lncRNAs) are transcripts with sequences greater than 200 nucleotides, but are not able to be translated into protein [14]. Accumulating evidence has suggested that numerous lncRNAs are aberrantly expressed in various tumors [15–17]. In addition, lncRNAs sometimes act as competing endogenous RNAs (ceRNAs) [18] or sponges for microRNAs (miRNAs) [19, 20], and then induce tumor biological behaviors. Exosomes are membrane-derived small vesicles with a size range of 20–150 nm [21] that contain various types of functional RNAs (e.g., lncRNAs, ceRNAs, miRNAs) and proteins. They are secreted by donor cells and taken up by other nearby cells [22]. Exosomes derived from tumor cells contribute to tumor progression through communication between tumor parenchyma and stroma [23–26]. However, whether lncRNAs released by exosomes cause EMT is not well understood.

LINC01133, a novel lncRNA, was first reported in 2015 to be highly expressed in lung squamous cell carcinoma (LSCC), but not in lung adenocarcinoma [27]. Recently, it has been verified that LINC01133 is involved in multiple types of malignant tumors, such as LSCC, osteosarcoma [20], cervical cancer [28], hepatocellular carcinoma [29], colorectal cancer (CRC) [30], and gastric carcinoma [31]. These studies collectively suggested that LINC01133 is an important regulator of cell proliferation and tumorigenesis, and may be a potential therapeutic target. However, the expression of LINC01133 in PDAC exosomes and its detailed functions in PDAC metastasis remain to be studied. In addition, we hypothesized that pancreatic cancer cells (PCCs) could generate LINC01133-rich exosomes and delivered to PSCs and then promote PSC secreted more Periostin. Periostin would then in turn affect PCCs by making them produce more LINC01133-rich exosomes, which would finally lead to metastatic behaviors. We believe this forms a positive feedback loop.

EMT can be induced through a variety of signaling pathways, such as Wnt/β-catenin. However, the role of LINC01133 in the EMT process remains largely unknown. Axis inhibition protein 2 (AXIN2) was reported as an oncogene and participates in the regulation of cell proliferation, migration, and other functions in several human cancers [32]. More significantly, we ultimately elucidated that LINC01133-mediated EMT of PCCs resulted from its negative regulation of AXIN2 expression. In this study, we show that LINC01133 promotes EMT and metastasis by interacting with Enhancer Of Zeste Homolog 2 (EZH2), and then promotes trimethylation of H3K27. This further silences AXIN2 and suppresses GSK3 activity, and ultimately promotes activation of the Wnt/β-catenin signaling pathway. Taken together, this study provides unique insights into the biological functions and potential mechanisms exerted by LINC01133 in PDAC development and progression.

## Results

### LncRNA expression profile in pancreatic cancer

**Fig. 1:**
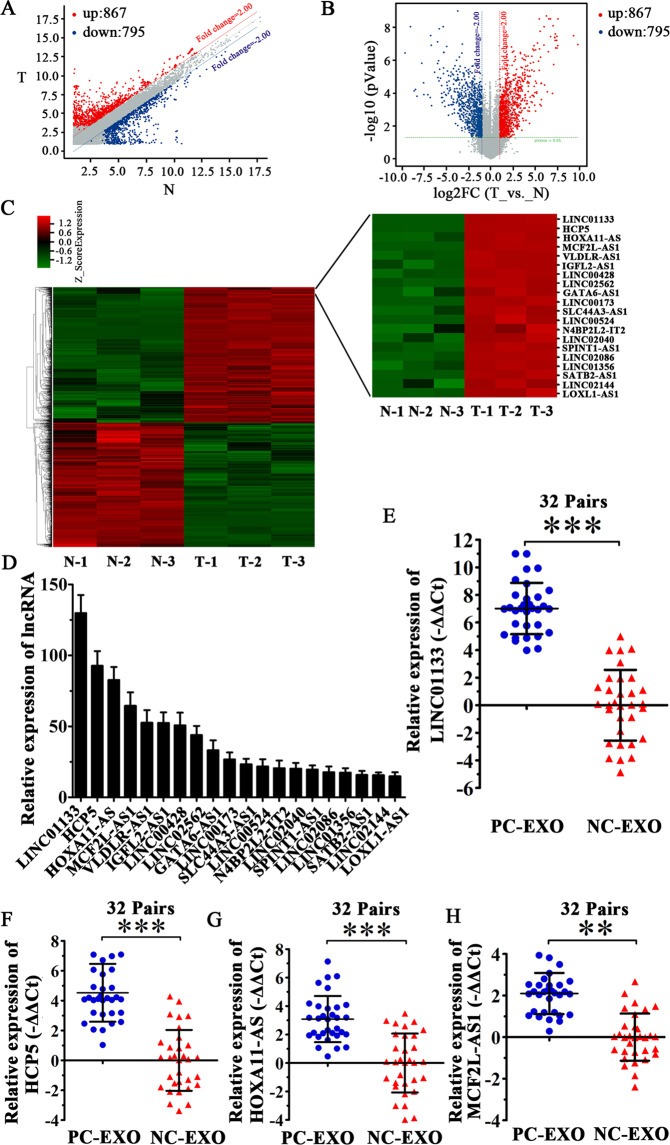
Identification of lncRNA expression profiles in pancreatic cancer. **A** Scatter plot of expression of various lncRNAs in the exosomes of pancreatic cancer cells and normal pancreatic ductal epithelial cells. The lncRNAs above the top red line and below the bottom blue line are those upregulated or downregulated more than twofold. T: pancreatic cancer cell exosomes; N: normal pancreatic ductal epithelial cell exosomes. **B** Volcano plot depicting the differential expression between these two groups. The red and blue dots represent lncRNAs with more or less than twofold changes in expression, respectively. **C** Hierarchical cluster analysis of the top 20 upregulated lncRNAs in pancreatic cancer cell exosomes matched with normal pancreatic ductal epithelial cell exosomes. **D** Histogram depicting the relative expression of the top 20 upregulated lncRNAs in pancreatic cancer cell exosomes compared with normal pancreatic ductal epithelial cell exosomes. **E–H** We detected the expression of top four upregulated lncRNAs in samples from 32 pairs pancreatic cancer tissues by qRT-PCR. All experiments were repeated three times, and the significance was analyzed using a Student’s *t* test. Data are shown as mean ± SD (**P < 0.01 and ***P < 0.001 vs. control).

Differences in lncRNA expression profiles between PCC exosomes (SW1990-exo) and normal pancreatic ductal epithelial cell exosomes (HPDE-exo) were detected by high-throughput human lncRNA microarrays through fold change (FC) (Fig. 1A) and volcano plot (Fig. 1B). Differentially expressed lncRNAs are shown in Fig. 1C. The top 20 most differentially expressed lncRNAs in the exosomes of PCCs are shown in Fig. 1D, and the details are listed in Table 1. The relative expression levels of lncRNAs LINC01133 (Fig. 1E), HCP5 (Fig. 1F), HOXA11-AS (Fig. 1G), and MCF2L-AS1 (Fig. 1H) in samples from 32 PDAC patients compared with their normal adjacent tissue were examined by qRT-PCR. Therefore, we selected the most highly expressed lncRNAs in PCCs exosomes and pancreatic cancer tissues simultaneously using high-throughput human lncRNA microarrays.

**Table 1:** Top 20 upregulated lncRNAs in pancreatic cancer.

lncRNA	*P* value	Regulation	FC (abs)	Chromosome	Length	GeneSymbol	GeneID
hsa_bnc053409	0.0000000082	Up	129.85507	1	1138	LINC01133	100505633
hsa_bnc053745	0.0000000035	Up	92.6851	6	2539	HCP5	10866
hsa_bnc050064	0.0000841120	Up	82.70869	7	1549	HOXA11-AS	221883
hsa_bnc052518	0.0000000092	Up	64.53599	13	1155	MCF2L-AS1	100289410
hsa_bnc050440	0.0000251281	Up	52.52521	9	887	VLDLR-AS1	401491
hsa_bnc059602	0.0002492875	Up	52.3898	19	1644	IGFL2-AS1	645553
hsa_bnc058247	0.0000264479	Up	50.69584	13	596	LINC00428	104355147
hsa_bnc062853	0.0000040678	Up	43.903297	4	553	LINC02562	105377283
hsa_bnc055243	0.0012384561	Up	33.162422	18	1788	GATA6-AS1	100128893
hsa_bnc051486	0.0000002072	Up	26.752466	12	1597	LINC00173	100287569
hsa_bnc055614	0.0003383940	Up	23.298841	1	1331	SLC44A3-AS1	101928079
hsa_bnc058135	0.0000094308	Up	21.798803	14	418	LINC00524	338002
hsa_bnc051198	0.0070255282	Up	20.543621	13	4890	N4BP2L2-IT2	116828
hsa_bnc124528	0.0007485811	Up	20.224829	3	366	LINC02040	102724763
hsa_gnc061524	0.0000097710	Up	19.571608	15	780	SPINT1-AS1	102724362
hsa_gnc143903	0.0000021493	Up	17.67771	17	3929	LINC02086	105371809
hsa_bnc055392	0.0004379514	Up	17.361753	1	1824	LINC01356	100996702
hsa_bnc051128	0.0000005782	Up	15.722862	2	3316	SATB2-AS1	150538
hsa_bnc135157	0.0045535015	Up	15.677369	5	530	LINC02144	105379069
hsa_gnc053666	0.0000075834	Up	14.800527	15	2179	LOXL1-AS1	100287616

*FC* fold change.

### LINC01133 expression is significantly upregulated in PCCs and exosomes

Expression levels of the top five highly expressed lncRNAs (LINC01133, HCP5, HOXA11-AS, MCF2L-AS1, VLDLR-AS1) were separately verified in four PCC lines (SW1990, CFPAC-1, AsPC-1, and Panc-1) and one normal pancreatic ductal epithelial cell line (HPDE) by qRT-PCR. The levels of these lncRNAs in exosomes extracted from these cells were also examined. The results confirmed that LINC01133 is the most highly expressed lncRNA in both PCCs and their secreted exosomes, especially in CFPAC-1 and SW1990 cells (Supplementary Fig. 1A/B). Therefore, LINC01133 was chosen to explore the function of exosomal lncRNAs in pancreatic cancer. We constructed siRNAs and a lentiviral vector for silencing and stable overexpression of LINC01133, respectively, in CFPAC-1 and SW1990 cells. The overexpression and silencing efficiencies are shown in Supplementary Fig. 2, and we selected one siRNA fragment with the best silencing effect for the experiment.

### Periostin promotes exosomes secretion by PCCs to induce EMT

**Fig. 2:**
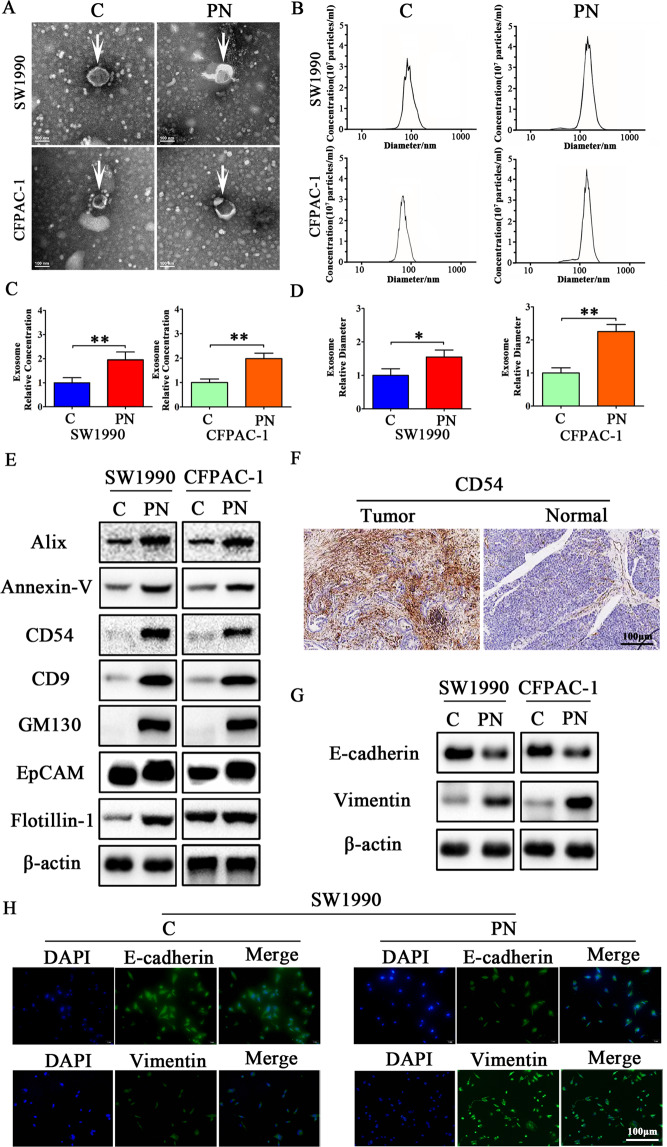
Periostin promotes exosome secretion from pancreatic cancer cells that induce EMT. **A** Electron microscopy images of exosomes isolated from SW1990 and CFPAC-1 cells treated with or without human recombinant Periostin protein (PN). **B** Concentration and size analysis of SW1990-exo and CFPAC-1-exo isolated by ultracentrifugation with or without PN. **C**, **D** Concentration and diameter analysis of SW1990-exo and CFPAC-1-exo treated with or without PN. The experiment was repeated three times, and the significance was analyzed using a Student’s *t* test. Data are shown as mean ± SD (*P < 0.05 and ** *P* < 0.01 vs. control). **E** Western blot analysis for exosomal proteins Alix, Annexin-V, CD54, CD9, GM130, EpCAM, and Flotillin-1 treated with or without PN in SW1990 and CFPAC-1 cells. β-actin was used as a loading control. **F** Expression of exosomal CD54 was examined by IHC in PDAC tissues. **G** The EMT effect was further confirmed by western blot analysis of epithelial or mesenchymal markers in SW1990 and CFPAC-1 cells treated with or without PN. **H** The EMT effect was validated by immunofluorescence analysis in SW1990 cells treated with or without PN.

To determine the effect of Periostin on exosomes released from PCCs, SW1990 and CFPAC-1 cells were co-cultured with human recombinant Periostin protein (PN) (1 μg/ml). Exosomes were isolated after 48 h and then photographed by electron microscopy and quantitated by nanoparticle tracking analysis (NTA). As shown in Fig. 2A/B, typical exosome particles were observed. NTA revealed that exosomes from SW1990 and CFPAC-1 cells harvested from the PN group demonstrated significantly higher nanoparticle concentrations (1.95-fold, *P* < 0.01, and 1.98-fold, *P* < 0.01; Fig. 2C) and size distribution (1.55-fold, *P* < 0.05, and 2.25-fold, *P* < 0.01; Fig. 2D) compared with the control group. As shown in Fig. 2E, increased exosome marker expression levels of Alix, Annexin-V, CD54, CD9, GM130, EpCAM, and Flotillin-1 were observed in SW1990 and CFPAC-1 cell exosomes after being co-cultured with PN for 48 h. In addition, CD5 expression was clearly higher in pancreatic cancer tissues than in the normal adjacent tissues by IHC (Fig. 2F). Thus, these results suggested that Periostin increased exosome secretion by PCCs. After SW1990 and CFPAC-1 cells were co-cultured with PN for 48 h, expression level of the epithelial cell marker E-cadherin was decreased, while expression level of the mesenchymal cell marker Vimentin was increased (Fig. 2G). Consistent with the above observations, the immunofluorescence assay suggested that SW1990 and CFPAC-1 cells extended many cellular pseudopods and cell morphology changed from condensed to spindle shaped. This was accompanied by increased expression of Vimentin and decreased expression of E-cadherin (Fig. 2H). Taken together, these data indicate that Periostin promotes exosome secretion by PCCs, which then induces EMT in vitro.

### The positive feedback interaction between periostin and exosomal LINC01133 is associated with advanced pathologic stage of PDAC

**Fig. 3:**
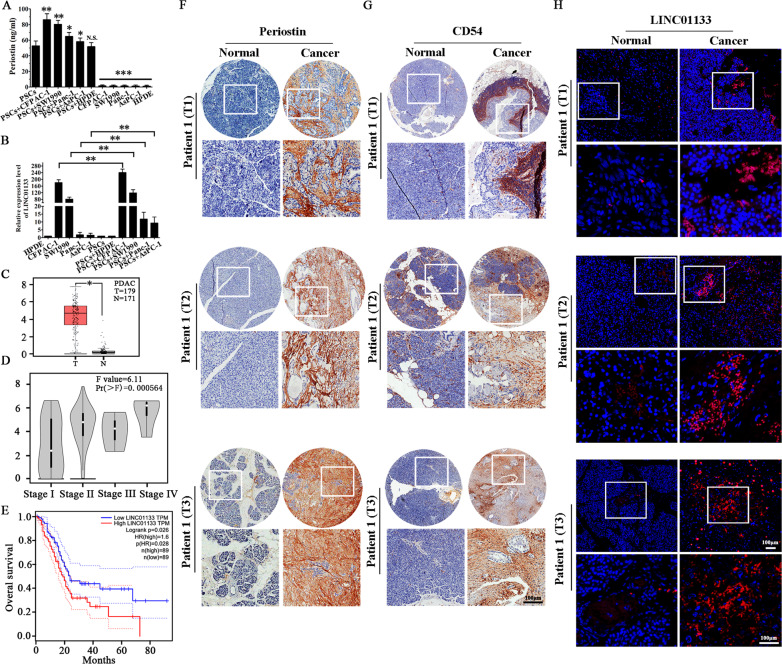
Increased LINC01133 expression positively correlates with Periostin and exosomal CD54 expression, as well as with poor PDAC patient survival. **A** Periostin expression in PSCs was enhanced when PSCs were co-cultured with PCCs. The experiment was repeated three times, and the significance was analyzed by a Student’s *t* test. Data are shown as mean ± SD (*P < 0.05, **P < 0.01 and ***P < 0.001 vs. PSCs). **B** LINC01133 expression in PCCs was also enhanced when PCCs were co-cultured with PSCs. The experiment was repeated three times, and the significance was analyzed using a Student’s *t* test. Data are shown as mean ± SD (**P < 0.01, PCCs vs. PSCs + PCCs.). **C** TCGA database analysis suggested that LINC01133 had significantly higher expression in PDAC tissues (*P* < 0.05). **D** TCGA database analysis also suggested that LINC01133 was significantly positively corelated with the TNM stage of PDAC patients (*P* < 0.001). **E** High expression of LINC01133 was significantly positively correlated with poor overall survival of PDAC patients, as suggested by TCGA database analysis (*P* = 0.028). **F**, **G** Immunohistochemical staining of 80 paired pancreatic cancer and matched normal tissues with anti-periostin and anti-exosomal CD54 antibodies and the representative patient samples of clinical stages T1, T2, and T3 are shown. **H** Representative images of LINC01133 expression (red, detected by RNA-FISH) in the same tumor tissue and adjacent non-tumor tissues.

When PSCs were co-cultured with four PCC lines (CFPAC-1, SW1990, Panc-1, AsPC-1) and HPDE cell line, Periostin expression was enhanced, as observed by ELISA (Fig. 3A). In addition, the expression of LINC01133 in PSCs + PCCs groups was increased compared with the PCCs separate culture, as detected by qRT-PCR (Fig. 3B). The clinicopathologic characteristics of 80 PDAC patients are listed in Table 2. We conducted analysis using The Cancer Genome Atlas (TCGA) database and found that LINC01133 expression was significantly higher in PDAC patients (Fig. 3C), positively correlated with tumor node metastasis (TNM) stage (Fig. 3D), and positively correlated with shortened patient overall survival (Fig. 3E). Next, we analyzed the expression levels of Periostin, exosomal CD54, and LINC01133 in the same tissues of 80 PDAC patients and their corresponding paired normal tissues by TMA and FISH, respectively (Fig. 3F–H). In addition, as shown in Table 3, LINC01133, Periostin, and CD54 are primarily expressed in PDAC tissues. Furthermore, LINC01133 expression was positively associated with Periostin and CD54 expression in the same patient tissue.

**Table 2:** The clinicopathologic correlations of LINC01133 expression in 80 pancreatic cancer patients.

Parameters	High expression (*n* = 68)	Low expression (*n* = 12)	*P*
Gender			0.8012
Male	37	7	
Female	31	5	
Age			0.3782
<60	36	8	
≥60	32	4	
pT stage			0.0015
T1	5	5	
T2	33	6	
T3	30	1	
pN stage			0.0011
N0	10	8	
N1	22	2	
N2	24	1	
N3	12	1	
Distant metastases			0.0203
No	32	10	
Yes	36	2

**Table 3:** Expression of LINC01133, Periostin, and CD54 in PDAC tissues.

		Periostin		CD54		P (LINC01133 vs Periostin)	P (LINC01133 vs CD54)
		+	−	+	−		
**LINC01133**	+	51	7	54	9	<0.01	<0.01
	−	13	9	9	8		

### Exosomal LINC01133 promotes proliferation, migration, invasion, EMT, and inhibits apoptosis in PDAC cells

**Fig. 4:**
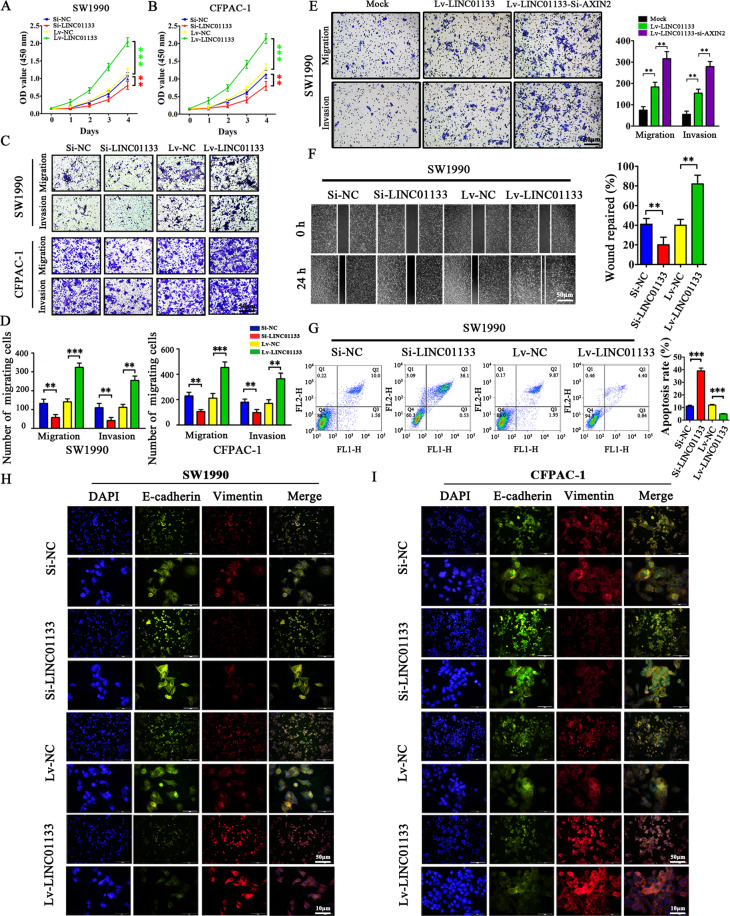
LINC01133 promotes pancreatic cancer cell proliferation, migration, invasion, and EMT, and decreases pancreatic cancer cell apoptosis. **A**, **B** LINC01133 knockdown decreased the proliferation rates of SW1990 and CFPAC-1 cells. In contrast, increased LINC01133 expression accelerated the proliferation rates of SW1990 and CFPAC-1 cells. OD values at 450 nm were measured by CCK-8 assay at 0, 1, 2, 3, and 4 days. The experiment was repeated three times, and the significance was analyzed by a Student’s *t* test. Data are shown as mean ± SD (**P < 0.01 and ***P < 0.001 vs. NC). **C**, **D** LINC01133 knockdown inhibited the migration and invasion rates of SW1990 and CFPAC-1 cells, whereas enhanced LINC01133 expression exerted the opposite effect. Cells were stained with crystal violet, and representative photographs of migratory or invading cells on the membrane coated with or without Matrigel (×50 magnification; Zeiss) are shown. The experiment was repeated three times, and the significance was analyzed by a Student’s *t* test. Data are shown as mean ± SD (**P < 0.01 and *** *P* < 0.001 vs. NC). **E** The migration and invasion ability were enhanced in AXIN2 silenced Lv-LINC01133 SW1990 cells. The experiment was repeated three times, and the significance was analyzed by a Student’s *t* test. Data are shown as mean ± SD (**P < 0.01 vs. Mock). **F** Migration activity was measured by wound-healing assay and the migration distance was measured from five random fields captured at each indicated time point. The experiment was repeated three times, and wound repair percentage of each cell line is shown in bar charts as mean ± SD (**P < 0.01 vs. NC). **G** Apoptosis was evaluated in SW1990 transfected with Si*-*NC, Si-LINC01133, Lv-NC and Lv-LINC01133 by Annexin-V-PI staining and flow cytometry. The apoptosis experiment was repeated three times, and the representative histograms are shown. The apoptosis rates are shown as mean ± SD (***P < 0.001 *vs*. NC). **H**–**I** The EMT effect was further validated by immunofluorescence analysis in SW1990 and CFPAC-1 cells.

To further assess the role of exosomal LINC01133 in PDAC, we constructed a LINC01133-overexpressing lentiviral vector (Lv-LINC01133) and one LINC01133-targeting siRNA (Si-LINC01133). After CFPAC-1 and SW1990 cells were transfected with Lv-LINC01133 lentivirus or Si-LINC01133 siRNA, we measured SW1990 and CFPAC-1 cell growth by Cell Counting Kit-8 (CCK-8) assay. The results suggested that LINC01133 could significantly increase cell proliferation (Fig. 4A/B). Likewise, LINC01133 led to a marked enhancement of cell migration and invasion, as seen with transwell assays (Fig. 4C/D). We next used siRNAs to silence AXIN2 in Lv-LINC01133 SW1990 cells and found the migration and invasion abilities to be clearly enhanced (Fig. 4E). We further detected the cell migration ability by wound-healing assays. As shown in Fig. 4F, the wound repairing rate in LINC01133-overexpressing SW1990 cells was significantly enhanced, and cell mobility was decreased following knockdown of LINC01133. Next, we conducted flow cytometry experiments to investigate the role of LINC01133 in cell apoptosis. We observed that the percentage of apoptotic SW1990 cells induced by Si-LINC01133 was higher compared with Si-NC (39.07% *vs*. 11.10%, *P* < 0.001). In contrast, the percentage of apoptotic SW1990 cells induced by Lv-LINC01133 was decreased compared with Lv-NC (4.92% vs. 11.96%, *P* < 0.001). These results suggested that LINC01133 overexpression could significantly decrease rates of cell apoptosis (Fig. 4G). Importantly, we found that LINC01133 overexpression could also promote EMT in PDAC cells. Therefore, we analyzed the expression of EMT markers in SW1990 and CFPAC-1 cells by immunofluorescence. We found that the expression of E-cadherin decreased and the expression of Vimentin increased in LINC01133 overexpression cells. Conversely, knockdown of LINC01133 increased E-cadherin expression and decreased Vimentin expression in these cell lines (Fig. 4H/I). Taken together, these data suggest that exosomal LINC01133 can promote proliferation, migration, invasion, and EMT, as well as inhibit apoptosis, in PDAC cells.

### Exosomal LINC01133 promotes PDAC tumor growth and EMT in vivo and facilitates the peritoneal metastasis of PCCs

**Fig. 5:**
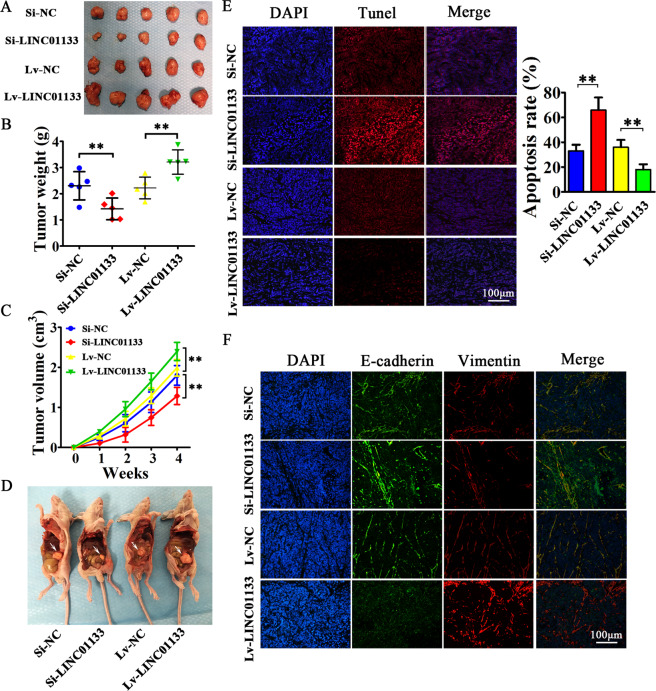
Exosomal LINC01133 can promote the tumorigenicity of pancreatic cancer cells in vivo, as well as EMT. **A** 4 × 10^6^ CFPAC-1 cells treated with Si-LINC01133, Si-NC, Lv-LINC01133, or Lv-NC were injected into the right side of nude mice. A month later, the mice were sacrificed, and all tumor grafts were excised. **B** CFPAC-1 cells treated with Lv-LINC01133 exhibited increased weight of xenografts. In contrast, CFPAC-1 cells treated with Si-LINC01133 showed reduced weight of xenografts (**P < 0.01 vs. NC). **C** CFPAC-1 cells treated with Lv-LINC01133 exhibited quicker growth and increased tumor volume. In contrast, CFPAC-1 cells treated with Si-LINC01133 showed slower growth and reduced tumor volume of xenografts (**P < 0.01 vs. NC). **D** 1 × 10^6^ CFPAC-1 cells treated with Si-LINC01133, Si-NC, Lv-LINC01133 or Lv-NC were respectively injected into the lower left quadrant of the abdomen and the dissemination ability in the abdominal cavity was evaluated. The metastatic nodules are marked by white arrowheads. **E** LINC01133 significantly inhibited apoptosis in xenograft tumors of CFPAC-1 cells as shown by TUNEL assay. The apoptosis rate was counted in five random fields and the experiment was repeated three times. Data are shown as mean ± SD (**P < 0.01 vs. NC). **F** Expression of E-cadherin and Vimentin in xenograft tumors was then analyzed by immunofluorescence.

We then performed xenograft tumor assays to explore the effect of exosomal LINC01133 on tumor progression in vivo. Stably down/upregulated LINC01133 CFPAC-1 cells and their corresponding control cells were subcutaneously injected into nude mice. They were sacrificed on day 28 after inoculation and the xenograft tumors were extracted (Fig. 5A). We found that knockdown of LINC01133 in PCCs significantly decreased tumor growth and reduced tumor weight and volume in nude mice compared with the Si-NC group. In contrast, increased tumor weight and size were observed in the LINC01133 overexpression group compared with the Lv-NC group (Fig. 5B/C). In addition, there were more obviously visible and larger peritoneal metastatic nodules in LINC01133 upregulation group compared with the NC group (Fig. 5D). Next, we used a TUNEL assay to investigate the effects of LINC01133 expression on tumor apoptosis rates. The results suggested that apoptosis rates were significantly increased in Si-LINC01133 group and decreased in Lv-LINC01133 group when compared with their corresponding control groups (66% vs. 33% in the Si-LINC01133 group; 18% vs. 36% in the Lv-LINC01133 group, respectively; *P* < 0.01) (Fig. 5E). Immunofluorescence staining showed that Vimentin expression was decreased and E-cadherin expression was increased when LINC01133 was knocked down, while overexpression of LINC01133 had the opposite results (Fig. 5F). Collectively, LINC01133 could promote tumor growth and EMT in vivo and facilitate peritoneal metastasis, which provides adequate conditions for PDAC progression and metastasis.

### Interaction of exosomal LINC01133 with AXIN2

**Fig. 6:**
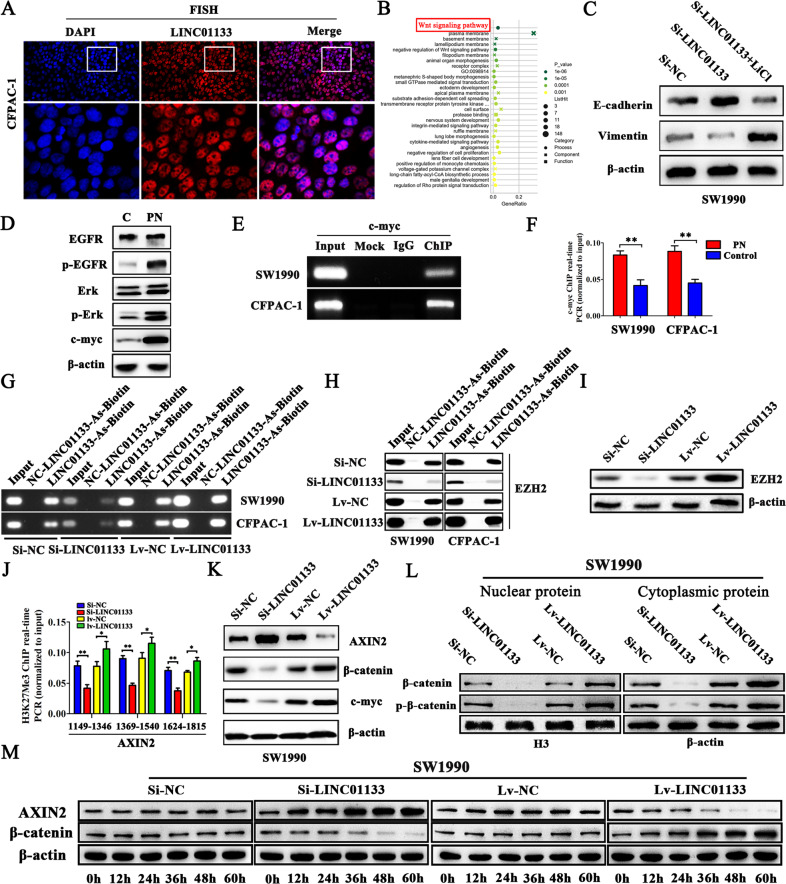
LINC01133 promotes EMT through the Wnt/β-catenin signaling pathway. **A** The FISH results suggested that LINC01133 transcripts were more abundant in the nucleus than in the cytoplasm. **B** The LINC01133-associated gene is mainly enriched in the Wnt pathway by KEGG analysis. **C** The expression change of E-cadherin and Vimentin proteins after adding LiCl in Si-LINC01133 transfected SW1990 cells. **D** Periostin can promote EGFR-Erk-c-myc signaling. **E** ChIP-PCR showed that c-myc occupied the LINC01133 promoter region. **F** The binding of c-myc at LINC01133 promoter region was enhanced by PN stimulation by ChIP-qPCR. The experiment was repeated three times, and the significance was analyzed by a Student’s *t* test. Data are shown as mean ± SD (**P < 0.01 vs. control). **G** ChIRP was used to examine the association of LINC01133 and biotin probes in different LINC01133 treatment groups. **H** ChIRP was used to examine the association of LINC01133 and EZH2 in different LINC01133 treatment groups. **I** Protein levels of EZH2 were positively associated with LINC01133 expression. **J** ChIP-qPCR suggests that LINC01133 promotes H3K27Me3 occupation of the AXIN2 promoter regions. The experiment was repeated three times, and the significance was analyzed by Student’s *t* test. Data are shown as mean ± SD (**P < 0.01, *P < 0.05 vs. NC). **K** LINC01133 was negatively related with AXIN2 and positively related with β-catenin and c-myc. **L** Downregulated LINC01133 triggered a reduction of nuclear and cytoplasmic β-catenin and p-β-catenin (Ser675) protein levels. H3 was used as the nuclear control, and β-actin was used as the cytoplasmic control. **M** The time course elucidated Si-LINC01133 and Lv-LINC01133 induces or inhibits AXIN2 expression at about 36 h and β-catenin can be upregulated or downregulated by Lv-LINC01133 and Si-LINC01133 also at about 36 h.

The FISH experimental results revealed that LINC01133 transcripts were more abundant in the nucleus than in the cytoplasm (Fig. 6A). To further explore the exact molecular mechanisms of exosomal LINC01133 in PDAC, KEGG analyses were performed. We found that LINC01133 was positively associated with the Wnt pathway (Fig. 6B). In addition, Wnt signaling agonist lithium chloride (LiCl), which is widely used to activate Wnt/β-catenin signaling, was added into 1640 medium to generate the Si-LINC01133 + LiCl group. E-cadherin protein expression levels in the Si-LINC01133 group was increased, while Vimentin levels were decreased. Conversely, the Si-LINC01133 + LiCl group showed increased Vimentin and decreased E-cadherin protein expression levels, as shown in Fig. 6C. Therefore, the LINC01133-associated gene in PDAC is mainly enriched in the Wnt pathway. Compared with the control group, the PN-treated group led to a significant increase in p-EGFR, p-Erk, and c-myc expression (Fig. 6D). ChIP assays suggested that c-myc could bind to the LINC01133 promoter region (Fig. 6E). ChIP-qPCR analysis showed that c-myc binding activity was much higher in the PN-treated group than in the control group (Fig. 6F). Collectively, these results showed that LINC01133 could be regulated by Periostin via EGFR pathway activity.

**Fig. 7:**
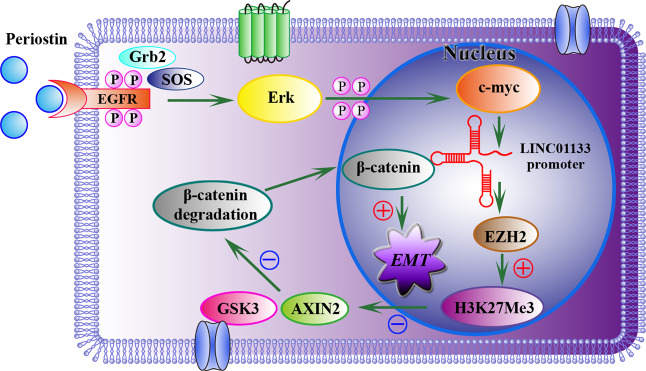
The mechanism diagram of LINC01133 promotes EMT through Wnt/β-catenin Pathway regulated by Periostin. LINC01133 can be up-regulated by Periostin via EGFR pathway, and LINC01133 interacts with EZH2 and then promotes H3K27 trimethylation which further silences AXIN2 and suppresses GSK3 activity, ultimately activating β-catenin.

ChIRP results showed that LINC01133 could be pulled down with biotinylated probes, and was enriched with EZH2 compared with the control (Fig. 6G/H). Moreover, EZH2 was positively correlated with LINC01133 (Fig. 6I). To understand how LINC01133 regulated Wnt through EZH2, we performed ChIP-qPCR assays to determine the exact regulatory mechanism. We designed three paired primers targeting the promoter region of AXIN2. As seen in Fig. 6J, we found that silencing of LINC01133 attenuated the interaction between H3K27Me3 and AXIN2, while overexpression of LINC01133 markedly improved it. Next, we found AXIN2 was increased in LINC01133 knockdown group and decreased in LINC01133 overexpression group. Conversely, β-catenin and c-myc levels were lower in LINC01133 knockdown group and higher in LINC01133 overexpression group (Fig. 6K). Besides, downregulated LINC01133 triggered a reduction of nuclear and cytoplasmic β-catenin and p-β-catenin (Ser675) protein levels (Fig. 6L). As shown in Fig. 6M, Si-LINC01133 and Lv-LINC01133 induces or inhibits AXIN2 expression, respectively, at about 36 h. In addition, β-catenin can be upregulated or downregulated by Lv-LINC01133 and Si-LINC01133, respectively, also at about 36 h. The mechanism diagram is shown in Fig. 7. Taken together, ChIP-qPCR assays showed that c-myc occupied the LINC01133 promoter region, and that Periostin could promote c-myc expression. This helps elucidate why Periostin can promote LINC01133 expression. In addition, our results suggest that LINC01133 may regulate the activity of PCCs through Wnt/β-catenin signaling pathway. Therefore, we clarified a new mechanism of exosomal LINC01133 on PDAC EMT in vitro and in vivo, which may be an extremely key factor affecting PDAC progression.

## Discussion

In recent years, exosomes have become one of the hot topics in the study of diagnostic biomarkers and therapeutic targets for human cancers. Multiple tumor cell-derived exosomes can circulate in the blood of patients, which may participate in the formation of tumor microenvironment and promote tumor development. LncRNAs have been increasingly recognized as functional regulators in cell differentiation and growth [33, 34]. In this study, we have determined that LINC01133 is overexpressed in PCCs exosomes. Further experiments suggested that exosomal LINC01133 can promote the proliferation, migration, invasion, EMT, and tumorigenesis of PCCs by epigenetically regulating AXIN2, which serves as a suppressor of Wnt/β-catenin pathway.

PSCs are mainly responsible for stroma production and tumor-stroma interactions. Previous studies have shown that Periostin is specifically secreted by PSCs [12, 35]. Periostin promoted the proliferation, metastasis, invasion, and clonality of PCCs, as well as tumor growth, in subcutaneous xenografts. To verify whether Periostin can promote the secretion of LINC01133, we used recombinant human PN to highly express Periostin in PCCs. We found that Periostin could significantly increase the concentration and diameter of exosomes. In addition, Periostin increased Vimentin expression and decreased E-cadherin expression significantly, suggesting that Periostin can promote EMT behavior in these cells. We believe that Periostin can promote exosome secretion by PCCs, thereby promoting LINC01133 expression.

We have also determined that LINC01133 has significantly higher expression levels in pancreatic cancer tissues compared with the matched adjacent tissues, which suggests that LINC01133 may act as a potential prognostic biomarker and therapeutic target for pancreatic cancer. However, a recent report showed that LINC01133 has low expression in CRC and indicated that LINC01133 acts as a tumor suppressor in this disease [30], which is in contrast to our findings in pancreatic cancer. The different functions of LINC01133 may be associated with different tissue types. Thus, LINC01133 expression appears to be tissue-specific and varies by cancer type. In addition, the biological mechanisms of LINC01133 are also varied in different tumors. For example, Yang et al. revealed that LINC01133 could inhibit gastric cancer progression by acting as a miR-106a-3p sponge [36]. Zeng et al. reported that LINC01133 promoted the proliferation and migration of human osteosarcoma cells by sponging miR-422a [20]. In this study, we have discovered that LINC01133 can influence Wnt/β-catenin signaling by epigenetically regulating AXIN2. KEGG analyses revealed that LINC01133 mainly influences this pathway. The Wnt/β-catenin signaling pathway plays a key role in cell development and tissue homeostasis [37].

Increasing studies have found that the dysregulation of Wnt/β-catenin signaling can cause many diseases, including prostate cancer [38], acute myeloid leukemia [39], breast cancer [40], lung adenocarcinoma [41], and CRC [42]. Interestingly, it has also been shown to be pivotal during pancreatic cancer progression, especially in its most common type, PDAC [43], by regulating cell proliferation, apoptosis, EMT, and other processes [44]. EMT is closely related to pancreatic cancer metastasis [23]. To date, there are few studies on the relationship between LINC01133 and Wnt signaling. In this study, we have clarified that the LINC01133-associated gene in PDAC is mainly enriched in the Wnt pathway. We also suggest that the dysregulation of the canonical Wnt signaling pathway is a result of alterations in LINC01133 expression related to EMT in pancreatic cancer.

More importantly, we have demonstrated that Periostin can increase p-EGFR, p-Erk, and c-myc expression, and c-myc can bind to the LINC01133 promoter region. Therefore, LINC01133 was regulated by Periostin mainly via EGFR pathway activity. Next, LINC01133 can interact with EZH2 and then promote trimethylation of H3K27, which can further silence AXIN2 and suppress GSK3 activity and finally inhibit degradation of β-catenin.

## Conclusions

We have shown the biological and clinical significance of exosomal LINC01133 in pancreatic cancer progression and suggested that LINC01133 was positive correlated with late TNM stage and poor overall survival in PDAC patients. We also clarified the underlying molecular mechanisms of LINC01133, which promoted the proliferation, migration, invasion, and EMT of PCCs. These findings elucidated that exosomal LINC01133 could be used as a therapeutic target for PDAC patients.

## Methods

### Cell culture and clinical specimens

Human PSCs were purchased from ScienCell research laboratory (Carlsbad, CA, USA) and maintained in stellate cell medium (ScienCell). Human PCC lines CFPAC-1, AsPC-1, Panc-1, and SW1990, as well as the HPDE cell line, were obtained from the Type Culture Collection of the Chinese Academy of Sciences. All cells were cultured at 37 °C in a 5% CO_2_ incubator.

Thirty-two fresh human pancreatic cancer samples and their adjacent tissues were collected from Shanghai First People’s Hospital with patients’ informed consent. All patients were pathologically diagnosed with PDAC and did not undergo radiotherapy or chemotherapy.

### Exosome isolation

CFPAC-1 and SW1990 cells (70–80% confluent) were cultured in RPMI 1640 medium with 10% exosome-depleted FBS (EXO-FBS-50A-1, System Biosciences, CA, USA) for 48 h. Exosomes were isolated from the cell culture supernatant by ultracentrifugation using Total Exosome Isolation Reagent (Invitrogen, Carlsbad, CA, USA) according to the manufacturer’s instructions. The size distribution and concentrations of exosomes were measured by NanoSight NS500 System (Nanosight Ltd, Malvern, England). Exosomes were observed by transmission electron microscopy (JEOL JEM1400, Japan) using the copper mesh suspension method. In addition, expression levels of representative exosome-related markers Alix, Annexin-V, CD54, CD9, GM130, EpCAM, and Flotillin-1 were detected by western blot (see below).

### Microarray

Microarray analysis was performed in the laboratory of OE Biotechnology Company in Shanghai, People’s Republic of China. Total RNA isolated from the CFPAC-1 and SW1990 cell exosomes was quantified using a NanoDrop ND-2000 instrument (Thermo Fisher Scientific, Waltham, MA, USA) and the integrity was assessed using an Agilent Bioanalyzer 2100 instrument (Agilent Technologies, Palo Alto, CA, USA). Sample labeling, microarray hybridization, and washing were performed based on the manufacturer’s standard protocols.

### Cell transfection

The lentivirus suspension used for LINC01133 overexpression was purchased from GenePharma Co., Ltd (Shanghai, China) and named Lv-LINC01133. The scrambled negative control was named Lv-NC. The expression of LINC01133 was measured by qRT-PCR. Three LINC01133 stealth siRNAs (siRNA #1, siRNA #2, and siRNA #3), three AXIN2 stealth siRNAs (siRNA 1#, siRNA 2#, and siRNA 3#), and a negative control siRNA were designed and synthesized by GenePharma Co., Ltd. (Supplementary Tables 1 and 2). The silencing efficiency of each siRNA was verified by qRT-PCR after 48 h of transfection. We selected the best silencing siRNA for cell transfection and named it Si-LINC01133 or Si-AXIN2, and named the negative control siRNA Si-NC. Cells were harvested for subsequent experiments.

### Cell proliferation, migration/invasion, and apoptosis assays

Cell proliferation was assessed with a CCK-8. A total of 3 × 10^3^ cells in different groups (Si-NC, Si-LINC01133, Lv-NC, and Lv-LINC01133) were seeded in 96-well plates and then treated with CCK-8 reaction fluid. Cell proliferation was measured using a Safire2 microplate reader (Tecan, Switzerland). Absorbance at 450 nm was recorded at 0, 24, 48, 72, and 96 h time points.

Transwell assays (Corning, Tehama, CA, USA) were used to analyze the migration and invasion capacities of SW1990 and CFPAC-1 cells. For migration assays, different groups (Si-NC, Si-LINC01133, Lv-NC, or Lv-LINC01133) of SW1990 (5 × 10^4^) and CFPAC-1 (3 × 10^4^) cells in 200 μL serum-free medium were separately seeded in the upper chambers of the plate. The lower chambers were then filled with 400 μL of medium containing 20% FBS. The cells were incubated for another 48 h at 37 °C, then any migrated cells were imaged and counted by microscopy. For invasion assays, Matrigel (BD Biosciences, Franklin Lakes, NJ, USA) was precoated on the upper chambers of the plate to simulate the in vivo extracellular matrix. Different groups (Si-NC, Si-LINC01133, Lv-NC, or Lv-LINC01133) of SW1990 (1 × 10^5^) and CFPAC-1 (6 × 10^4^) cells in 200 μL serum-free medium were added to the upper chambers. The remaining procedures were performed as done in the migration assay. Next, we used siRNAs to silence AXIN2 in Lv-LINC01133 SW1990 cells to determine if they could influence the induced migration and invasion of the cells. In the migration or invasion assays, different groups (Mock, Lv-LINC01133 and Lv-LINC01133-si-AXIN2) of SW1990 (3 × 10^4^ or 6 × 10^4^) cells in 200 μL serum-free medium were separately seeded in the upper chambers. The remaining procedures were performed as done previously.

Wound-healing assays were also used to observe cell migration capacities. The cell monolayers were scratched with 200 μL pipette tips and photographed at 0 and 24 h by inverted light microscopy.

Flow cytometry was used to analyze apoptosis in vitro. The procedures suggested by the Annexin-V-FITC Apoptosis Kit (BD Biosciences) were followed. Cell samples were incubated with Annexin-V and Propidium iodide (PI) at room temperature for 30 min. Viable apoptotic cells can be found in the lower right quadrant, while non-viable apoptotic cells can be found in the upper right upper quadrant. These experiments were carried out using a CytoFLEX flow cytometer (Beckman Coulter, Inc, USA). All experiments were repeated three times independently.

### Tissue microarray (TMA) construction

TMAs containing 80 pancreatic cancer samples and their matched adjacent tissues were used in this study (Outdo Biotech, Shanghai, China). Immunohistochemical staining for Periostin, CD54, and LINC01133 were assessed and scored by two pathologists.

### Immunofluorescence

EMT gene expression, both in vitro and in vivo, was analyzed by immunofluorescence. Mouse anti-E-cadherin antibody (CST, 14472) and Goat anti-Vimentin antibody (CST, 5741) were used. The experimental steps were included in Supplementary materials. An Olympus BX-43 microscope and Image J software were used to analyze the data.

### Western blot

Proteins in cell lysates were separated by the manufacturer’s standard protocols. Primary antibodies included exosomal markers: Mouse anti-Alix (CST, 2171), Rabbit anti-Annexin-V (CST, 8555), Rabbit anti-CD54 (CST, 4915), Rabbit anti-CD9 (CST, 13174), Rabbit anti-GM130 (CST, 12480), Rabbit anti-EpCAM (CST, 2626), and Rabbit anti-Flotillin-1 (CST, 18634); EMT markers: Mouse anti-E-cadherin (CST, 14472), and Rabbit anti-Vimentin (CST, 5741); and Wnt/β-Catenin pathway markers: Rabbit anti-AXIN2 (CST, 2151), Rabbit anti-β-catenin (CST, 8480), Rabbit anti-Phospho-β-catenin (Ser675) (CST, 4176) and Rabbit anti-c-myc (CST, 5605). All the abovementioned antibodies were diluted at 1:1000 in 1× Tris-buffered saline containing Tween-20 (TBST) at 4 °C overnight. Secondary antibodies included goat anti-rabbit IgG horseradish peroxidase (HRP)-conjugated secondary antibody or horse anti-mouse IgG HRP-conjugated secondary antibody (CST, 7074 or 7076) was diluted at 1:2000 in 1× TBST for 1 h at room temperature. Finally, antigens on the membrane were detected with enhanced chemiluminescense detection reagents (Roche, Basel, Switzerland).

### Tumor xenograft model and tumorigenicity assay

Twenty four-week-old male immunodeficient BALB/c nude mice (weight: approximately 20 g each) were obtained from Shanghai Slack Laboratory Animal Co. Ltd (Animal license number SCXK (Shanghai) 2012-0002) and housed under specific pathogen-free conditions. CFPAC-1 cells (4 × 10^6^ cells in 20 μL of cell suspension) transfected with Si-LINC01133, Si-NC, Lv-LINC01133, or Lv-NC were subcutaneously implanted into the back of nude mice. Mice were randomly divided into four groups (*n* = 5 mice/group). The tumor volume was examined weekly by a caliper and calculated with the following formula: volume = 0.5 × length × width^2^. The nude mice were euthanized when the tumor volume reached 1 cm^3^ in size (after approximately 4 weeks). These xenograft tumors were then harvested, dissected, weighed, and embedded in paraffin or fixed in 10% formalin.

### TdT-mediated dUTP nick end labeling (TUNEL) staining

TdT-mediated dUTP nick end labeling (TUNEL) staining was performed to evaluate cellular apoptotic rates in mice tumor samples. The experimental steps were included in Supplementary materials. Image J software was used to analyze the captured images.

### Fluorescence in situ hybridization (FISH)

The cells were hybridized with 300 μL of Cy3-labeled LINC01133 probe-containing hybridization solution (5′-CY3-TCTTCTACTCTTTACCTCCTCCCAACCAT-CY3-3′) overnight at 37 °C according to the manufacturer’s standard protocols. Images were obtained with a Nikon fluorescence microscope.

### Chromatin immunoprecipitation (ChIP) assay

ChIP experiments were performed using a ChIP Kit (Millipore EZ-Magna, Billerica, MA, USA) according to the manufacturer’s instructions. The antibody used was c-myc (CST). qRT-PCR was performed to measure the expression of LINC01133 in the samples. The primer sequences for LINC01133 were 5′-TGGGAAAGAGGTTGCAGT-3′ (F) and 5′-CCAAAGGGAAGCTAAGGAG-3′ (R).

### Chromatin isolation using RNA purification assay (ChIRP)

Chromatin isolation using RNA purification (ChIRP) was performed with a ChIRP Kit (BersinBi, Guangzhou, China) according to the suggested protocol. 3′ end Biotin-triethylene glycol-modified DNA probes targeting LINC01133 were constructed and synthesized. Trizol (Invitrogen, Carlsbad, CA, USA) was used to collect total RNA, and protein expression was analyzed by western blot. The sequences of the biotin probes are available in Supplementary Table 3.

### Statistical analysis

Statistical differences between groups were compared using an unpaired two-sided Student’s *t* test. The association between LINC01133 and its related gene expression in patient samples was analyzed by the Pearson correlation. All statistical analyses were performed using SPSS 16.0 software (SPSS, Chicago, IL, USA) and GraphPad Prism 5 (GraphPad Software Inc., San Diego, CA, USA). The data were presented as mean ± standard deviation from at least three independent experiments and a *P* value < 0.05 was considered statistically significant.

## Supplementary information

SUPPLEMENTAL MATERIAL
