# Supplementary material for: Tumor-derived exosomal long noncoding RNA LINC01133, regulated by Periostin, contributes to pancreatic ductal adenocarcinoma epithelial-mesenchymal transition through the Wnt/β-catenin pathway by silencing AXIN2
Source: Oncogene. 2021 Apr 6;40(17):3164–79. doi: 10.1038/s41388-021-01762-0 (PMC8084735; doi:10.1038/s41388-021-01762-0)
Supplement: Supplementary file 1 — SUPPLEMENTAL MATERIAL [file 41388_2021_1762_MOESM1_ESM.doc]

**Methods**

**Immunofluorescence**

EMT gene expression, both in vitro and in vivo, was analyzed by immunofluorescence. Mouse anti-E-cadherin antibody (CST, 14472) and Goat anti-Vimentin antibody (CST, 5741) were used. For in vivo analysis, dewaxed and hydrated mice tumor sections were heated to 95-100°C in a sodium citrate solution for 30 min for antigen retrieval. Sections were then incubated with 5% BSA for 30 min at room temperature. For in vitro analysis, cell samples were first fixed with 4% paraformaldehyde (PFA) for 10 min and then incubated with 5% BSA for 30 min. After blocking non-specific antigens with BSA, both cell samples and tumor sections were incubated with the abovementioned antibodies at a 1:100 diluent concentration. An Olympus BX-43 microscope and Image J software were used to analyze the data.

**TdT-mediated dUTP nick end labeling (TUNEL) Staining**

TdT-mediated dUTP nick end labeling (TUNEL) staining was performed to evaluate cellular apoptotic rates in mice tumor samples. After the mice were euthanized by cervical dislocation, tumors were removed and then processed into paraffin sections. Before staining, sections were dewaxed with xylene and hydrated with gradient concentrations of ethanol. The suggested procedures for the TUNEL Apoptosis Assay Kit (Roche, Mannheim, Germany) were followed. Briefly, sections were incubated with TUNEL working solution for 30 min at 37°C. Immunofluorescence staining was observed using an Olympus BX-43 microscope. Image J software was used to analyze the captured images.


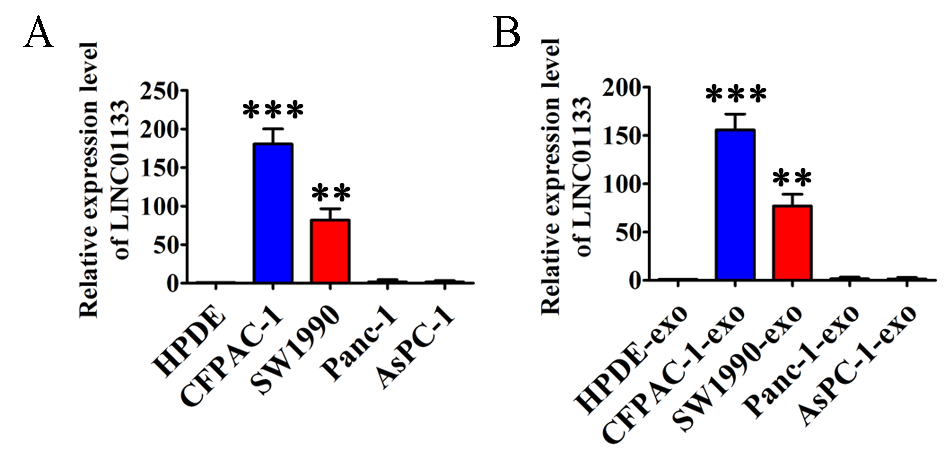


**Supplementary Fig.1** LINC01133 was verified in SW1990, CFPAC-1, AsPC-1 and Panc-1 and 1 normal pancreatic ductal epithelial cell (HPDE) **(A)** as well as their extracted exosomes **(B)** by Real-time PCR comparied to HPDE (***p*<0.01, ****p*<0.001).

**
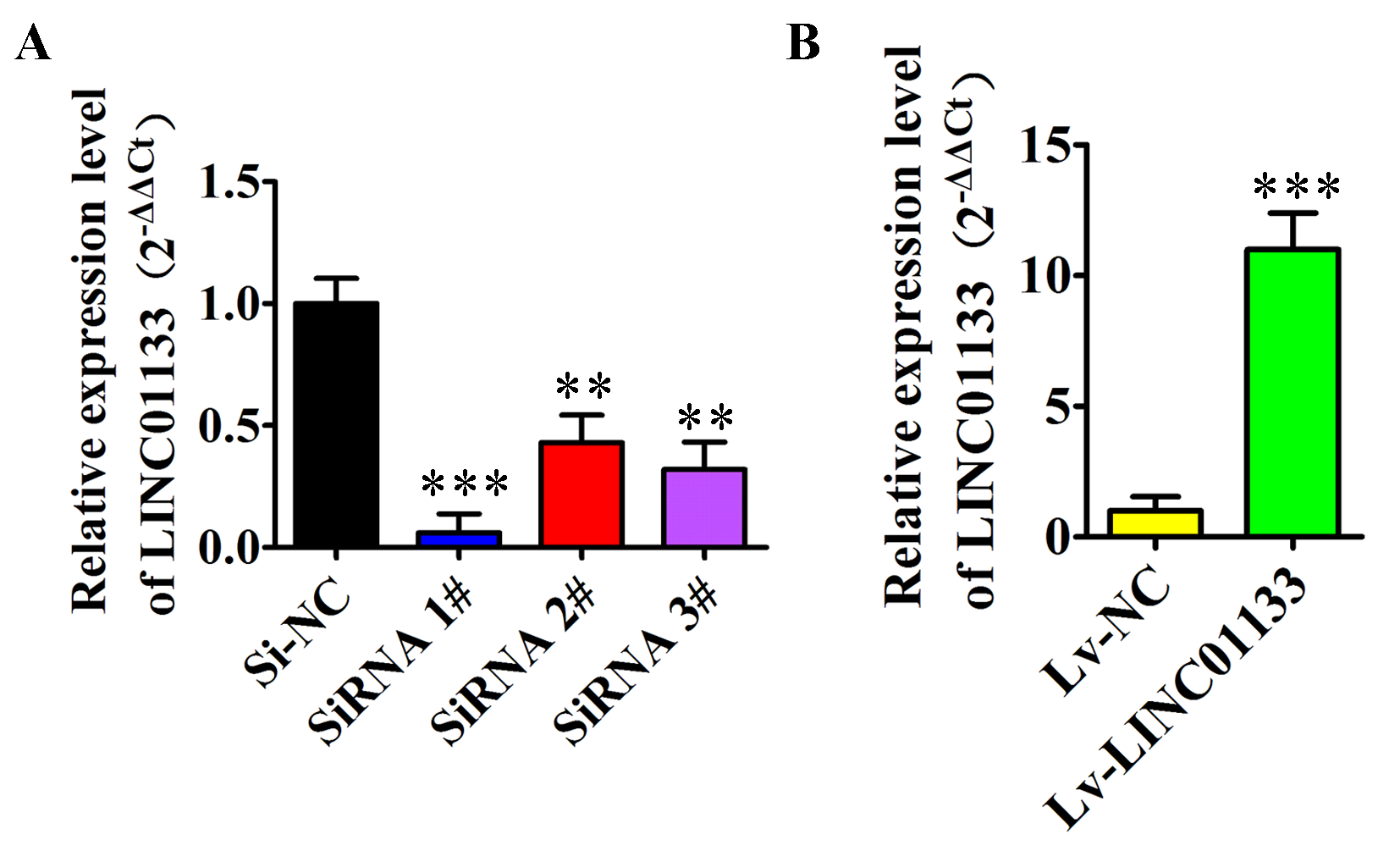
**

**Supplementary Fig.2A** Relative expression of LINC01133 in CFPAC-1 cells transfected with Si-NC, siRNA 1#, siRNA 2# and siRNA 3# as determined by qRT-PCR. **Fig.2B** Relative expression of LINC01133 in LINC01133-overexpressing CFPAC-1 cells. Data presented are the mean ± SD of three independent experiments (***p*<0.01, ****p*<0.001).

**Supplementary Table 1. The sequences of LINC01133 siRNA**

| Name | Sequences | |
| --- | --- | --- |
| siRNA 1# | | F: GGAGCCAUUAACAAAGCUU(dTdT)  R: AAGCUUUGUUAAUGGCUCC (dTdT) |
| siRNA 2# | | F: CCCAUUCCAUACCAUCUUU(dTdT)  R: AAAGAUGGUAUGGAAUGGG (dTdT) |
| SiRNA 3# | | F: GGCAUAGGGAUCCAUUUAU(dTdT) |
|  |  | R: AUAAAUGGAUCCCUAUGCC(dTdT) |
| Nega­tive control siRNA | | F: UUCUUCGAACGUGUCACGU(dTdT)  R: ACGUGACACGUUCGGAGAA(dTdT) |

**Supplementary Table 2. The sequences of AXIN2** siRNA

| Name | Sequences | |
| --- | --- | --- |
| siRNA #1 | | F: GUGGAUACCUUAGACUUCU(dTdT)  R: AGAAGUCUAAGGUAUCCAC (dTdT) |
| siRNA #2 | | F: CCGACUUCAAGUGCAAACU(dTdT)  R: AGUUUGCACUUGAAGUCGG (dTdT) |
| siRNA #3 | | F: GCGAUCCUGUUAAUCCUUA(dTdT) |
|  |  | R:UAAGGAUUAACAGGAUCGC (dTdT) |
| Nega­tive control siRNA | | F: UUCUUCGAACGUGUCACGU(dTdT)  R: ACGUGACACGUUCGGAGAA(dTdT) |

**[Supplementary Table](https://www.frontiersin.org/articles/10.3389/fcell.2019.00350/full#S10) 3. Sequences of biotin probes**

|  | Name | Sequences |
| --- | --- | --- |
| lnc4001376 | RiboTM h-linc01133(3bio)_ChIRP Probe_1 | TTACCACCACTGATGTCTAC |
| lnc4001377 | h-linc01133(3bio)_ChIRP Probe_2 | TCCATTCTCAGGTAACTTCA |
| lnc4001378 | h-linc01133(3bio)_ChIRP Probe_3 | TCTACTCTTTACCTCCTCCC |
| lnc4001379 | h-linc01133(3bio)_ChIRP Probe_4 | TGGTATGGAATGGGAGAATC |
| lnc4001380 | h-linc01133(3bio)_ChIRP Probe_5 | CTAAGGAGAAAGTTGGAGCA |
| lnc4001381 | h-linc01133(3bio)_ChIRP Probe_6 | GCTGTGGTGAGATTAGGAAA |
| lnc4001382 | h-linc01133(3bio)_ChIRP Probe_7 | TAAACAAACTCAGAGGCACT |
| lnc4001383 | h-linc01133(3bio)_ChIRP Probe_8 | TTTCCCAAGGCTGTTACATC |
| lnc4002118 | h-linc01133(3bio)_ChIRP Probe_9 | GCTGGAACAAGGACTTTCTG |
| lnc4002119 | h-linc01133(3bio)_ChIRP Probe_10 | TGGAATGGGAGAATCCATCC |
